# Trends in accidental poisoning and exposure to noxious substances involving drugs, medicaments, and biological substances–related deaths: A nationwide US analysis, 1999 to 2023

**DOI:** 10.1097/MD.0000000000048537

**Published:** 2026-05-01

**Authors:** Sujata Lodh, Muddassir Khalid, Amisha Kumari, Sumia Fatima, Rida Noor

**Affiliations:** a Department of Medicine, Dr. KNS Memorial Institute of Medical Sciences, Barabanki, India; b Department of Medicine, Nishtar Medical University, Multan, Pakistan; c Department of Medicine, Ghulam Muhammad Mahar Medical College, Sukkur, Pakistan; d Department of Medicine, Rawalpindi Medical University, Rawalpindi, Pakistan; e Department of Medicine, Bolan Medical College, Quetta, Pakistan.

**Keywords:** accidental poisoning, CDC, drugs, noxious substances, poisoning

## Abstract

Unintentional poisoning and exposure to harmful substances involving illicit drugs, medications other than those taken as prescribed, and biological substances have emerged as a leading cause of preventable death in the United States. The increase in drug-specific toxicity, particularly from synthetic opioids, has accelerated during the COVID-19 pandemic, thus highlighting a need for national surveillance to monitor emerging demographic and geographic patterns. The CDC WONDER Multiple Cause of Death database provided mortality data from 1999 to 2023. Deaths with accidental poisoning (International Statistical Classification of Diseases and Health-Related Problems–10th Revision codes X40–X49) coded as the underlying cause and poisoning due to drugs (T36–T50) as multiple causes were considered. Age-adjusted mortality rates were extracted from the SEER database (number of deaths per 100,000 individuals). They were analyzed according to sex, age strata, race/ethnicity, and geographic region via Joinpoint regression for estimation of *annual percentage change* and *average annual percentage change*, with statistical significance set at *P* < .05. From 1999 to 2023, all-ages age-adjusted mortality rates more than sextupled, from 4.8 (95% confidence interval: 4.7–4.9) to 30.8 (95% confidence interval: 30.6–31.0). The consistently higher male mortality culminated in 2022, where men had a crude mortality of 45.3 per million compared with 18.2 per million in women. The largest increase was from 2019 to 2020, after the start of the COVID-19 pandemic. It is most severe among middle-aged adults (ages 45–64) and is characterized by geographic disparities in mortality, with clustering in counties across the Appalachian region and Southern United States. Non-Hispanic Black and American Indian/Alaska Native people experienced the latest spikes in mortality. The ongoing rise in drug-related accidental poisonings indicates a growing, evolving epidemic in the United States. The findings identified a need for focused equity-based interventions targeting substance use treatment, social determinants of health, and enhanced surveillance to address the escalating burden of mortality due to drug toxicity.

## 1. Introduction

Accidental poisoning and exposure to toxic substances, namely environmental chemicals as well as medicine-related toxicological exposures, represent an increasing part of the global burden of disease (i.e., the combined effect on early death and illness-peripheral morbidity from health problems and damaging influences worldwide).^[1]^ Such instances are of particular concern when drug toxicity is implicated, as in the case of overdose with opioids, stimulants, or synthetic agents such as fentanyl. Thus, drug-related toxicity has intensified the burden in many regions, especially the United States, where synthetic opioids are at the heart of recent spikes.^[2–6]^

This issue is interlinked with broader health problems. Drug toxicity is not merely a standalone phenomenon; it overlaps with mental disorders, substance use, and other chronic conditions such as HIV, hepatitis, or cardiovascular diseases.^[7]^ In addition, unintended poisoning has risk factors similar to intentional injuries (e.g., suicide) and thus, a multiple-cause-of-death approach would be appropriate.^[6]^

Age plays a significant role. Although the risk of premature mortality is higher in older adults owing to polypharmacy and cognitive decline, young and middle-aged adults (18–45 years) often bear the brunt of mortality because of patterns of substance consumption, psychosocial stressors, and economic deprivation.^[2]^ Social determinants of health, including socioeconomic status, race, and sex, also play a role in disparities in poisoning mortality men experience, with racial minorities and low-income populations bearing the unequal burden from structural inequities that yield differences in healthcare education as well as social services access.^[8,9]^

Since the trends are complex and mortality is increasing, it is important to study the long-term trends using rigorous surveillance tools such as the Centers for Disease Control and Prevention Wide-ranging Online Data for Epidemiologic Research (CDC WONDER). This study aims to provide an overall national profile over time from 1999 to 2023 by analyzing deaths with accidental poisoning (International Statistical Classification of Diseases and Health-Related Problems–10th Revision [ICD-10] codes X40–X49) as the underlying cause, where drug toxicity (T36–T50) was listed as a multiple cause. These findings can guide innovative and targeted prevention, harm reduction, and policy development efforts aimed at reversing the trajectory of the epidemic of injury-related mortality. This study utilizes data from the CDC WONDER system, a publicly accessible mortality surveillance platform that provides nationwide death certificate data stratified by demographic and geographic characteristics. CDC WONDER is widely used in population-level epidemiologic research due to its standardized coding, national coverage, and reproducibility.

## 2. Materials and methods

### 2.1. Study sample

We used the CDC WONDER Multiple Cause of Death database to retrieve death files between 1999 and 2023 (provisional and partial included) for accidental poisoning and exposure to noxious substances, with these causes shown as the underlying cause of death (coded from ICD-10 codes X40–X49). Mortality data were extracted from CDC WONDER (https://wonder.cdc.gov) during September 2025 using the finalized multiple cause-of-death files available at that time. Also, drug-related toxic effects with (ICD-10 T36–T50) were listed as multiple causes to consider the role of different substances in such deaths. This publicly available database is maintained by the National Center for Health Statistics and includes death certificate data for US residents. Deaths were classified according to ICD-10 codes, recalling broad categories of drugs, medicaments, and biological substances rather than individual agents. Table 1 summarizes the substance categories included in the analysis (Table 1).

**Table 1:** ICD-10 categories of drugs, medicaments, and biological substances included in the analysis.

ICD-10 code range	Substance category	Examples
T36–T39	Non-opioid analgesics, antipyretics	Acetaminophen, salicylates
T40	Narcotics and psychodysleptics	Opioids, heroin, fentanyl
T41–T43	Anesthetics and psychotropic drugs	Sedatives, antidepressants
T44–T50	Other drugs and biological substances	Cardiovascular drugs, insulin
X40–X44	Accidental poisoning	Unintentional overdoses

ICD-10 = International Statistical Classification of Diseases and Health-Related Problems–10th Revision.

This study was exempt from local institutional review board approval because it used a de-identified government-issued public use dataset and followed the Strengthening the Reporting of Observational Studies in Epidemiology guidelines for reporting.

The database provides aggregated mortality statistics and is composed of the number of deaths and the age-adjusted death rates per 100,000 population based on the 2000 population. The data can also be broken down by sex, age category, race/ethnicity, and other demographics, facilitating more nuanced subgroup analysis.

### 2.2. Inclusion and exclusion criteria

All death certificates occurring from 1999 to 2023 with accidental poisoning (X40–X49) as the underlying cause of death and at least 1 drug toxicity code (T36–T50) among the multiple causes were included. Records with missing or excluded codes, or those which had suppressed or incomplete data, were not analyzed. There were no demographic restrictions (e.g., age, sex, or race/ethnicity or geographic region) in order to make findings generalizable to the broad national population.

### 2.3. Variables and measures

The data set included integrated mortality data (counts of deaths and age-adjusted death rates per 100,000 population) standardized to the year 2000 US standard population. Data were stratified by sex, age group, race/ethnicity, and region of residency to characterize demographic and temporal trends. Mortality data from 1999 to 2023 were obtained, and included deaths attributed to accidental poisoning and exposure to noxious substances (coded X40–X49 of ICD-10) for drug-related toxic effects (codes T36–T50 of ICD-10).

Age-adjusted mortality rates (AAMRs) per 100,000 population were reported with 95% confidence intervals (CIs) to control the statistical reliability. Key demographic characteristics were age (classified in 5 groups: infants and young children [0–4 years], school-aged children and adolescents [5–24 years], young adults [25–44 years], middle-aged adults [45–64 years], older adults [65–85+ years]), sex, race/ethnicity (non-Hispanic (NH) White, NH Black, Hispanic, NH American Indian/Alaska Native, Asian/Pacific Islander), and US Census-defined regions and states. Additional attributes such as place of death (home, hospital, hospice) and degree of urbanization were included whenever they were available to allow for more nuanced analysis. Urbanization data were available for the years 1999 to 2020 only and could accordingly not be compared beyond that period. Notably, deaths among the young adults (25–44 years) were only accessible up to 2020 at the point of extraction. Thus, trend analyses for this age group only covered the period 1999 to 2020.

The 2 time periods (1999–2020 and 2021–2023) provided a comparison of the recent present against longer-term trends. To examine trends over time, periods of rate change, and demographic differences in drug-involved accidental poisoning mortality in the United States, we utilized Joinpoint regression (JPR) analyses.

### 2.4. Statistical analysis

Mortality rates and demographic distributions were described with summary statistics over time. The Joinpoint Regression Program was used to examine the presence of statistically significant changes (“joinpoints”) in temporal trends of AAMRs. JPR analyses were performed using the National Cancer Institute Joinpoint Regression Program (version 5.0.2). Data extraction and preprocessing were conducted directly within the CDC WONDER platform without external modification. Annual percentage change together with the 95% CI, and attributable risk was estimated for each segment between joinpoints (where *P* < .05 indicates statistical significance). An average annual percentage change was calculated to provide a summary of the trend in the whole study period. Output parameters were lower and upper limits for the segment, annual percentage change, lower and upper CI values, and *P*-values. Microsoft Excel was used for data cleaning and descriptive analyses, and trend analyses were conducted in Joinpoint software. This study does not establish causality between specific substances and mortality outcomes. Causes of death were identified based on death certificate attribution using ICD-10 coding, reflecting associations rather than confirmed toxicological causation.

### 2.5. Ethical considerations

Given the de-identified, publicly available nature of CDC WONDER data, this secondary analysis was not subject to Institutional Review Board approval or informed consent. The data were collected and analyzed from September 10 to October 5, 2025.

## 3. Results

### 3.1. Analysis of overall and sex-stratified AAMRs

From 1999 to 2023, the AAMR increased notably for both sexes in the United States.

Males always showed higher mortality compared with females throughout the follow-up. The male rate in 1999 was 6.9 per 1,000,000 (95% CI: 6.8–7.1), and the female rate was 2.6 (95% CI: 2.6–2.7). This discrepancy continued to grow over the period and reached a peak in 2022, with the male rate at 45.3 (95% CI: 45.0–45.7) compared with a mean value of 18.2 (95% CI: 18.0–18.4) in females.

The overall trend in mortality rate showed a constant rise, from 4.8 (95% CI: 4.7–4.9) in 1999 to a peak of 31.8 (95% CI: 31.6–32.0) in the year 2022, with an estimated statistically significant increase per year (average annual percentage change = 1.5%). A marginal decrease was shown in 2023, where the rate had a slight reduction to 30.8 (95% CI: 30.6–31.0; Table S3, Supplemental Digital Content, https://links.lww.com/MD/R788).

The steepest year-over-year jumps took place from 2019 to 2020, coinciding with the start of the COVID-19 pandemic. During this period, male mortality rose from 29.0 (95% CI: 28.7–29.2) to 39.0 (95% CI: 38.7–39.3), and female mortality increased from 12.2 (95% CI: 12.0–12.4) to 15.8 (95% CI: 15.6–16.0). This surge is probably a result of a combination of accumulating stressors, diminished access to care, and increased use of substances throughout the pandemic.

The mean AAMR for the entire period was 19.7 (95% CI: 19.5–20.0) deaths among males and 9.0 (95% CI: 8.9–9.2) among females, and, considering all members of the population, it was 14.4 deaths per thousand people (95% CI: 14.3–14.5). These findings are consistent with previous studies showing similar mortality trends.

**Figure 1.**
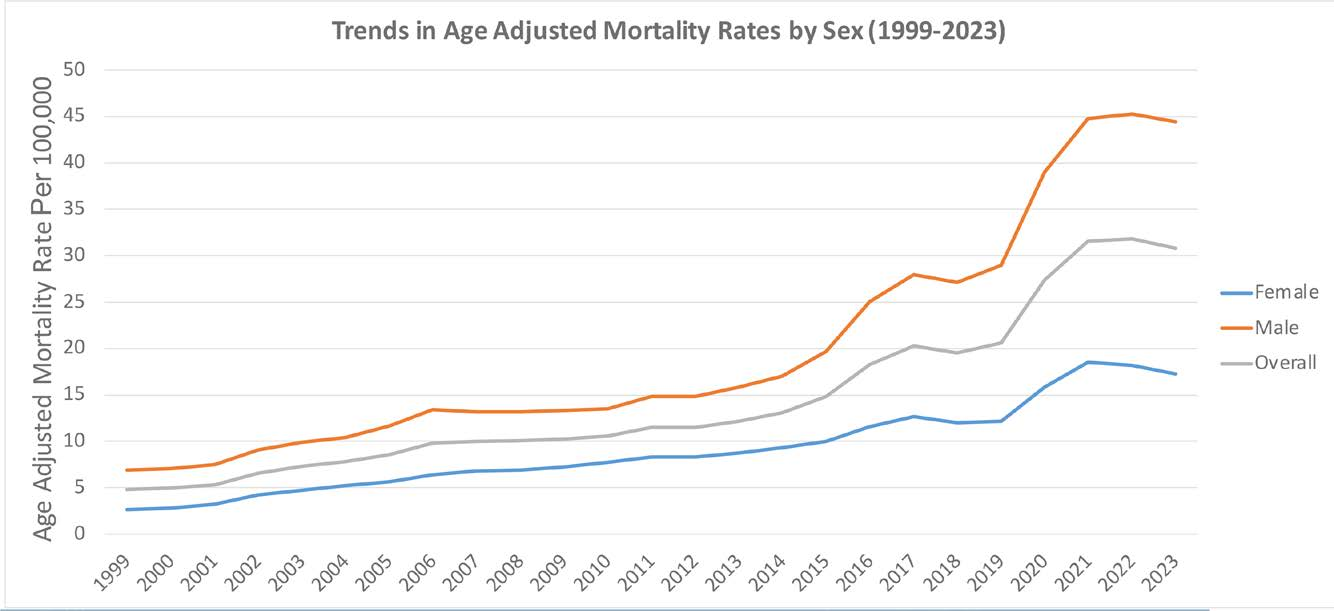
Sex-specific annual mortality trends due to accidental poisoning and exposure to noxious substances involving drugs, medicaments, and biological substances, 1999 to 2023; AAMRs per 100,000 US adults aged ≥25 years, stratified by sex. AAMR = age-adjusted mortality rate.

**Figure 2.**
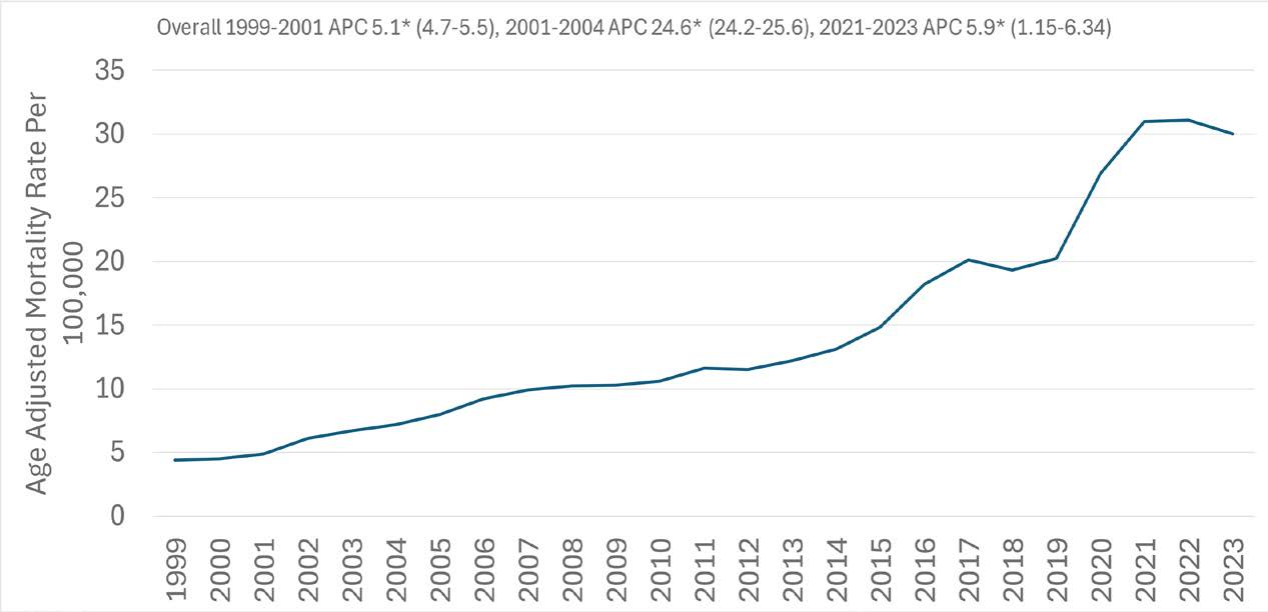
Overall annual mortality trends due to accidental poisoning and exposure to noxious substances involving drugs, medicaments, and biological substances, 1999 to 2023; overall AAMRs per 100,000 US adults aged ≥25 years. AAMR = age-adjusted mortality rate, APC = annual percentage change.

These trends are illustrated in Tables S1 and S4, Supplemental Digital Content, https://links.lww.com/MD/R788, and Figures 1 and 2.

### 3.2. Analysis of AAMRs stratified by age group

AAMR had different temporal and demographic characteristics across all age groups between 1999 and 2023. In general, the trend of mortality increased over age categories, with a steep increase in 2020 to 2021 occurring concurrently with COVID-19. These results are in line with international reports of excess mortality and disease burdens because of the pandemic.

#### 3.2.1. Infant and young (0–4 years)

From 1999 to 2023, the mortality rate among the population aged 0 to 4 was characterized by consistently low numbers. The AAMR ranged from 0.1 to 0.3 per 100,000 in 1999 to 2019, indicating stable levels of early childhood mortality trends. A slight increase was observed between 2020 and 2022, reaching a maximum of 0.6 (95% CI: 0.5–0.7) in 2022, followed by a slight decrease to 0.5 (95% CI: 0.4–0.6) in the year 2023. This category contributed to <7% of all deaths per annum during the study duration. It is likely that the observed variations represent improved neonatal and early childhood medical care, punctuated by temporary spikes during the time of the pandemic.

#### 3.2.2. School-aged and young adults (5–24 years)

The AAMR for those aged 5 to 24 years, begins at 1.2 in 1999 and continues to increase slowly until it reaches a value of approximately 5.0 (95% CI: 4.8–5.1) in the year 2019; then there is a rapid escalation to its highest value of about 7.7 (95% CI: 7.5–7.9) during the year 2020. The highest rate was 8.0 (95% CI: 7.8–8.1) in 2021, and then this went down to 6.2 (95% CI: 6.0–6.4) by the end of the projection period in 2023. As of 2021, the percentage of overall deaths in this age group increased from 1.4% in 1999 to 18.3%, possibly reflecting pandemic-related behavioral, educational, and mental health effects on adolescents and young adults. The 15 to 24-year age group was analyzed separately to capture early adult risk patterns for accidental poisoning, which differ from those observed in older adult populations.

#### 3.2.3. Young adults (25–44 years)

The 25 to 44 age bracket also saw some of the sharpest spikes in mortality over the period. The AAMR increased from 8.8 (95% CI: 8.6–9.0) in 1999 to 36.8 (95% CI: 36.4–37.2) in 2019, with a marked peak of up to 49.4 (95% CI: 48.9–49.8) in 2020. This increased trend probably results from the combinatorial effect of lifestyle diseases, substance use, and socioeconomic stressors, which were exacerbated due to the pandemic. This group accounted for about 11% of all deaths in 2020, which represented a striking escalation in the death profile of young adults.

#### 3.2.4. Middle-aged adults (45–64 years)

Mortality rates among middle-aged adults continued to rise throughout the study period. The AAMR increased from 6.5 (95% CI: 6.3–6.7) in 1999 to 32.8 (95% CI: 32.4–33.2) in 2019, with a pandemic-related increase to a level of 42.3 (95% CI: 41.8–42.7) in the year of COVID-19 (2020). The rate increased continually and peaked at 52.0 (95% CI: 51.5–52.6) in 2022, then plateaued at 51.9 (95% CI: 51.4–52.4) in the year 2023, while remaining at the same level from year to year. The rate of death in this group rose from 1.2% to 16.9%, which is consistent with reports associating midlife mortality rates with chronic metabolic and cardiovascular conditions and adverse COVID-19 outcomes.

#### 3.2.5. Older adults (65–85+ years)

The highest absolute increases and relative increases were seen among older people. It increased continuously from 2.9 (95% CI: 2.8–3.1) in 1999 to reach 8.3 (95% CI: 8.0–8.5) in 2020 and then expanded further to present at a level of 13.1 (95% CI: 12.8–13.4) by 2023. The percentage of total deaths in this age range rose from 2.4% to 18.9%, which highlights the manifest asymmetry in susceptibility to infectious and noncommunicable diseases among elderly people.

Between all groups, the mean AAMR was also lowest among infants and young children (0.20 per 100,000) and highest among middle-aged (29.2 per 100,000) and young adults (29.1 per 100,000). The general pattern exhibits a clear age-specific gradient, with the highest relative increases observed in 2020 to 2021, reflecting the worldwide influence of the COVID-19 pandemic on mortality trends.

**Figure 3.**
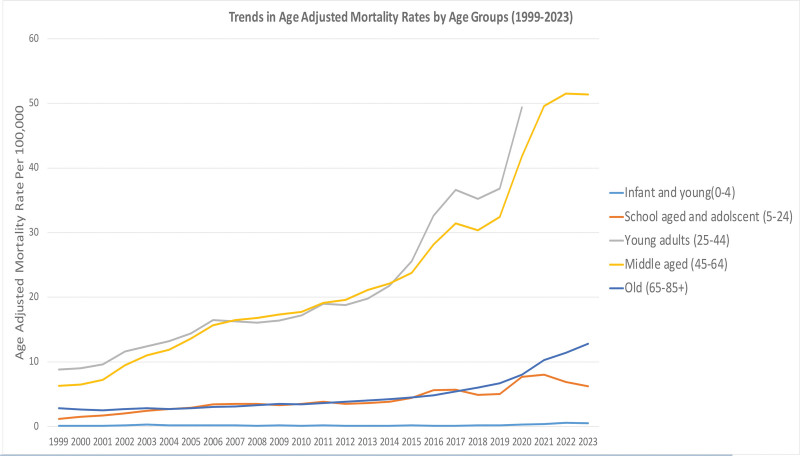
Age disparities in mortality due to accidental poisoning and exposure to noxious substances involving drugs, medicaments, and biological substances, 1999 to 2023; AAMRs per 100,000 US adults aged ≥25 years, stratified by age. AAMR = age-adjusted mortality rate.

These trends within each age group are presented in Figure 3.

### 3.3. Analysis of AAMRs stratified by race

Overall, between 1999 and 2023, we observed noticeable racial differences in AAMR by race (Table S5, Supplemental Digital Content, https://links.lww.com/MD/R788).

At the start of the study period, mortality was highest among NH Black individuals (7.6 per 1,000,000 [95% CI: 7.3–7.9]), followed by Hispanic or Latino (5.5 [95% CI: 5.3–5.8]) and NH White (4.6 [95% CI: 4.5–4.7]) populations. In NH American Indian or Alaska Native (AI/AN) and NH Asian or Pacific Islander groups, rates were relatively low at 4.2 (95% CI: 3.4–5.1) and 0.9 (95% CI: 0.7–1.1), respectively. The increases in death rates were consistent across all racial groups during the early 2000s, which was a mirror of what was happening nationwide: an increased use of prescription opioids. AAMRs were estimated to be 11.9 for NH Whites by 2010 (95% CI: 11.7–12.0), which was higher compared to all other groups, with AAMRs of 7.8 for NH Blacks (95% CI: 7.5–8.0), 12.9 among Hispanics (95% CI: 12.6–13.2) and the AAMR for NH AI/AN at a rate of 9.6 (95% CI: 8.6–10.5).

After 2015, the mortality gap widened precipitously as the medical market for pain pills transitioned to illicit drugs such as synthetic fentanyl. NH White and NH Black groups experienced the largest increases, growing from 16.7 (95% CI: 16.5–16.8) and 11.5 (95% CI: 11.2–11.8) in 2015 to 32.7 (95% CI: 32.5–33.0) and 42.2 (95% CI: 41.6–42.8) in 2021, respectively. In 2023, AAMR was highest among NH Blacks (47.5; 95% CI: 46.9–48.2), followed by NH AI/AN (42.0; 95% CI: 40.1–44.0) and NH Whites (30.5; 95% CI: 30.3–30.7). In contrast, NH Asian or Pacific Islander experienced the lowest mortality rates consistently below 5.5 per 1,000,000 throughout all years of study. Persons of Hispanic or Latino ethnicity had generally low rates with a peak at 6.7 (95% CI: 6.4–6.9) in the year 2023, indicative of partial protection due to cultural, social, and community-related effects that are referred to as the “Hispanic paradox.”

**Figure 4.**
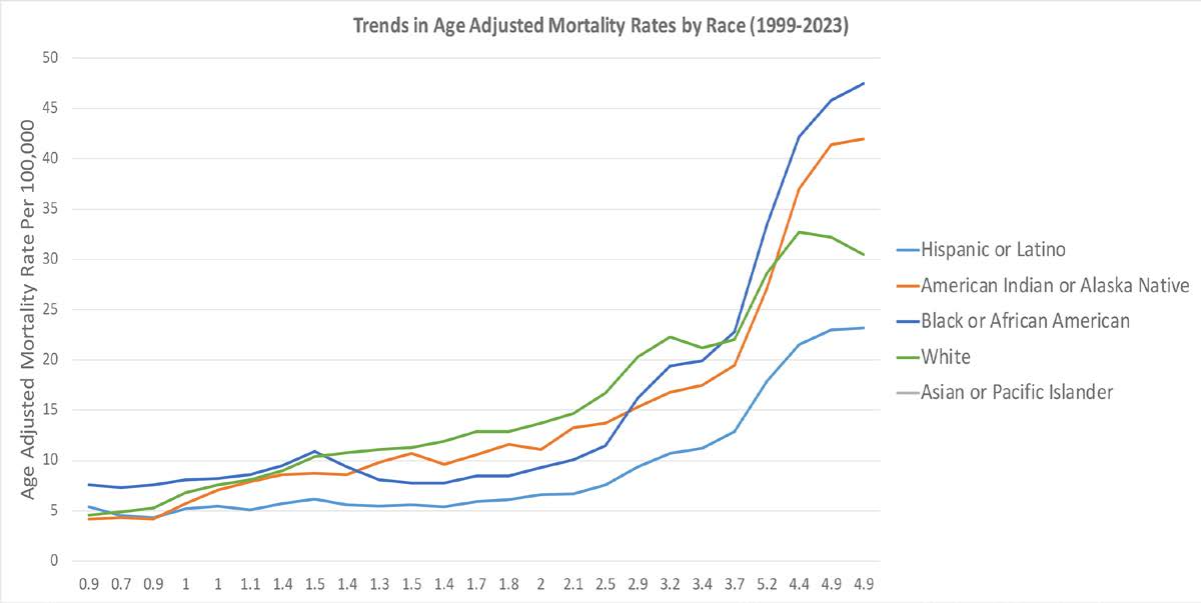
Racial and ethnic disparities in mortality due to accidental poisoning and exposure to noxious substances involving drugs, medicaments, and biological substances, 1999 to 2023; AAMRs per 100,000 US adults aged ≥25 years, stratified by race/ethnicity. AAMR = age-adjusted mortality rate.

These effects are shown in Figure 4 and summarized in Tables S1 and S5, Supplemental Digital Content, https://links.lww.com/MD/R788.

### 3.4. Analysis of deaths by place of occurrence

**Figure 5.**
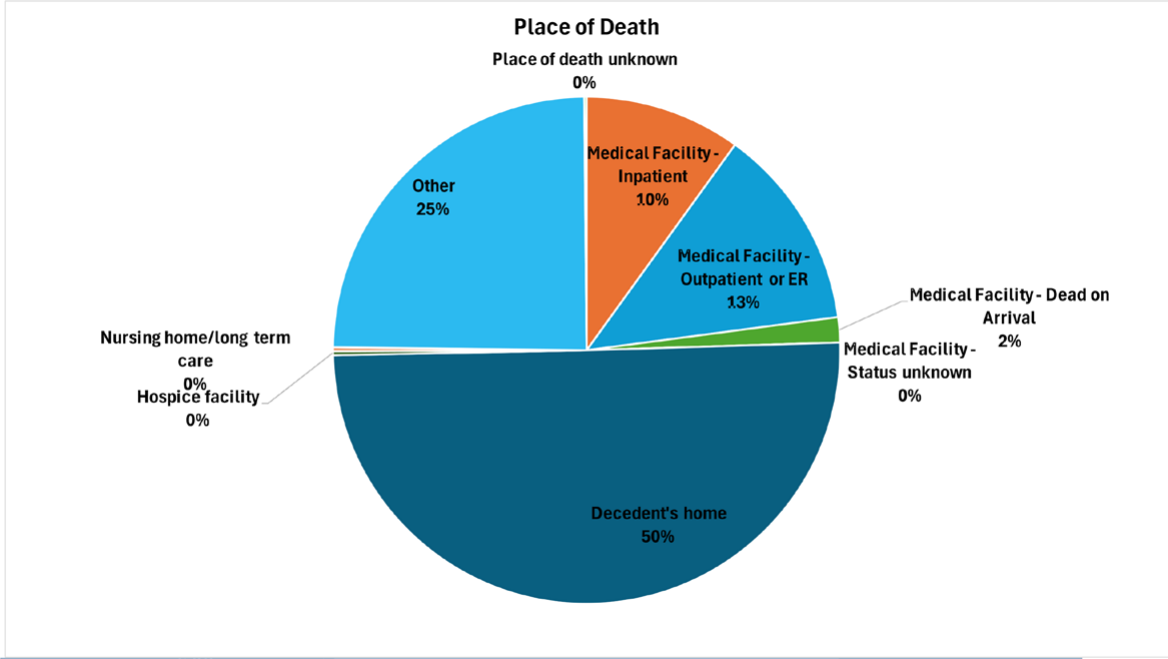
Place of death disparities in mortality due to accidental poisoning and exposure to noxious substances involving drugs, medicaments, and biological substances, 1999 to 2023; AAMRs per 100,000 US adults aged ≥25 years, stratified by place of death. AAMR = age-adjusted mortality rate.

Table S2, Supplemental Digital Content, https://links.lww.com/MD/R788, and Figure 5 illustrate the distribution of these deaths by place of death.

During 1999 to 2023, there were 1,118,830 older people who died from accidental poisoning and exposure to noxious substances related to drugs, medications, and biological substances in the United States. On analyzing the place of death, the highest number of deaths occurred at home, constituting 50.15% (n = 565,384) of deaths.

The proportion of deaths in hospitals was 27.4% (n = 289,179). Of those, 9.94% of deaths in the included hospitalized patients (n = 113,546), and 12.95% were in outpatients or emergency room settings (n = 153,082). Lower percentages were observed for dead-on-arrival (1.6%) and facility status unknown (<0.01%).

A large proportion of cases was from noninstitutional settings, highlighting the difficulty in controlling medication-related poisonings that are not in an acute care setting. Apart from deaths at home, 24.65% (n = 255,978) of deaths were discovered “others,” defined as public places, vehicles, and unknown locations. Deaths in nursing homes or long-term care facilities and hospice centers were relatively rare, at 0.25% each. Of the deaths, only 0.15% (n = 2581) did not report a site of occurrence, suggesting strong data completeness.

### 3.5. Geographic variation and temporal changes in AAMRs

Between 1999 and 2023, substantial geographic variation was observed in AAMRs.

#### 3.5.1. National overview

During the early period (1999–2020), state-specific AAMRs ranged from 5.3 deaths per 1,000,000 in Nebraska to 27.3 in West Virginia, indicating pronounced interstate disparities. By the later period (2021–2023), these differences had widened considerably, with rates ranging from 10.5 (95% CI: 9.6–11.4) in Nebraska to 83.3 (95% CI: 80.7–85.9) in West Virginia.

#### 3.5.2. Regional patterns

The Appalachian region, including West Virginia, Kentucky, Tennessee, and Ohio, consistently demonstrated the highest mortality rates in both study periods. West Virginia recorded the greatest increase, rising from 27.3 (95% CI: 26.8–27.8) to 83.3 (95% CI: 80.7–85.9), followed by Tennessee (16.5 → 53.8) and Kentucky (20.5 → 51.2). These patterns align with prior reports of opioid and polysubstance use being concentrated in rural and economically disadvantaged regions.

In contrast, Midwestern and Mountain states such as Nebraska, Iowa, South Dakota, and Montana maintained comparatively lower mortality throughout the study period, though all exhibited modest increases post-2020. The Northeastern states (e.g., Connecticut, Delaware, Rhode Island, and Maine) showed moderate-to-high baseline rates with sharp post-pandemic surges, particularly in Delaware (17.9 → 52.5) and Maine (14.7 → 49.8).

Southern and Western states also experienced marked escalation, notably Louisiana (14.6 → 50.9), California (9.7 → 26.8), and Arizona (15.7 → 36.2). Coastal states such as Florida (15.0 → 33.8) and Washington (11.8 → 33.6) reflected similar upward trajectories, underscoring the widespread impact across demographic and geographic lines.

**Figure 6.**
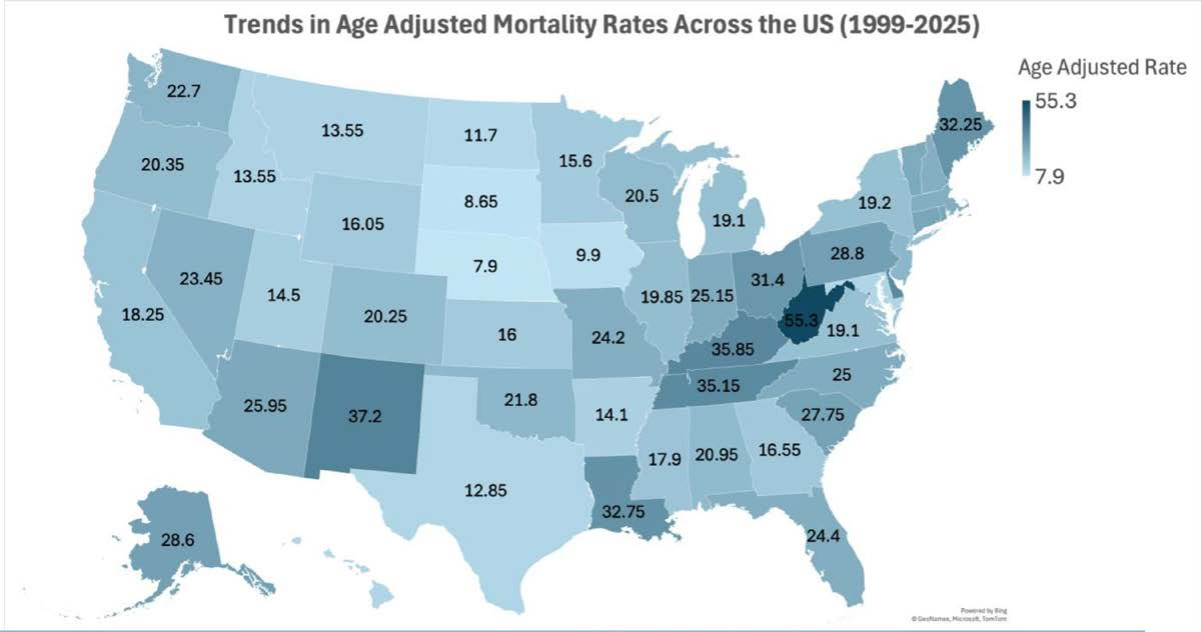
US state-level disparities in mortality due to accidental poisoning and exposure to noxious substances involving drugs, medicaments, and biological substances, 1999 to 2023; AAMRs per 100,000 US adults aged ≥25 years, stratified by US states. AAMR = age-adjusted mortality rate.

These findings are presented in Tables S6 and S7, Supplemental Digital Content, https://links.lww.com/MD/R788, and visualized in Figure 6.

### 3.6. Analysis of AAMRs stratified by urbanization

**Figure 7.**
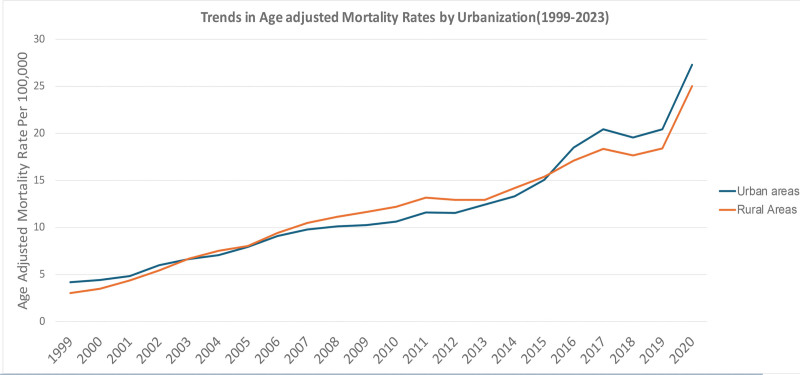
Urban–rural disparities in mortality due to accidental poisoning and exposure to noxious substances involving drugs, medicaments, and biological substances, 1999 to 2023; AAMRs per 100,000 US adults aged ≥25 years, stratified by metropolitan status. Urbanization-wise data are limited by CDC WONDER and could not be attained beyond the year 2020. AAMR = age-adjusted mortality rate, CDC WONDER = Centers for Disease Control and Prevention Wide-ranging Online Data for Epidemiologic Research.

AAMRs for unintentional poisoning/exposure to noxious substances involving drugs, medicaments, and biologicals increased considerably in urban and rural areas from 1999 through 2020 during the study period (Table S8, Supplemental Digital Content, https://links.lww.com/MD/R788). In 1999, AAMR was 4.2 (95% CI: 4.1–4.4) per million population in urban areas and 3.1 (95% CI: 2.8–3.3) in rural areas; from 2003 through to 2010 rural mortality exceeded that for urban populations (10.7; 95% CI: 10.4–10.9) with a high of 12.2 (95% CI: 11.7–12.7). Following 2015, urban places experienced a sharper mortality increase (mirroring the rise of synthetic opioids such as fentanyl), leading to 2020 AAMRs of 27.3 (95% CI: 26.8–27.7) for urban and 25.1 (95% CI: 24.4–25.8) for rural populations, respectively. The overall AAMR during the period were similar by urbanization – 11.9 (95% CI: 11.6–12.2) in urban and 11.8 (95% CI: 11.3–12.2) in rural areas – but differences over time, with rural areas leading in the early part of the epidemic and urban area at later years, illustrate how drug overdose has evolved geographically across the United States (illustrated in Fig. 7).

## 4. Discussion

This study shows that the AAMRs of accidental poisoning and exposure to noxious substances related to drugs, medicaments, and biological substances have soared from 1999 to 2023 in all demographic groups of populations in the United States, with an extremely high rate increase since 2019. The results are consistent with earlier national reports of a dramatic increase in drug overdose deaths during and after the onset of the COVID-19 pandemic, when healthcare delivery was disrupted, isolation from others took hold, and psychological stressors rose, accentuating the risk for use/relapse to substance use.^[10,11]^

The difference in death rates between the sexes was consistently larger for men and reflected behavioral and epidemiological evidence that men more frequently engage with high-risk drug use, have a lower frequency of health-seeking behaviors, and are exposed to higher levels of occupational or psychosocial stressors. They highlight the urgent need for targeted public health interventions, especially among men, who are far more likely to die due to drug poisoning. Continued research is needed to more fully understand the impact of socioeconomic, behavioral, and policy-related factors on these trends.^[12,13]^

Similarly, age-stratified patterns showed that although deaths continued to be low in children, the rates increased substantially in young and middle-aged adults, illustrating the persistent toll of the synthetic opioid epidemic on persons during their most productive years. The relatively sustained increase in elderly age also suggests that the risks of polypharmacy, comorbidities, and accidental drug interactions may not have declined significantly and raises questions on geriatric pharmacovigilance and community surveillance.^[14]^

Significant racial and regional disparities remain, with recent highs in mortality for NH Black and American Indian/Alaska Native populations, which have surpassed earlier phases of the opioid epidemic dominated by NH White communities. Collectively, these data indicate that despite an earlier predominance of the opioid crisis among NH White populations in some areas, the more recent surge in deaths among NH Black and AI/AN populations reflects a change in the affected population structures of the epidemic. Underlying structural inequalities, inequitable health care access, underdiagnosis of substance use disorders, and patterns of drug supply that differ by region are driving some of this evolving racial profile.^[15]^ That rates among NH Asian or Pacific Islander and Hispanic groups remain relatively low underscores the importance of social networks, family context, and potentially less exposure to high-potency synthetic opioids.^[16,17]^ This trend represents a disturbing broadening of the epidemic beyond its original demographic profile due to unjust gaps in access to care, stigma surrounding substance-use disorders, and the introduction of potent synthetic opioids, including fentanyl, into new drug markets.^[18,19]^ Among states in the Appalachian and Southern regions, in particular West Virginia, Kentucky, and Tennessee, experienced the sharpest increases as observed by prior analyses, with these same states being tied to economic deprivation, low capacity for addiction treatment services, high rates of prescription opioids (and methadone) prescribing rates – all correlate with sustained overdose mortality.^[20]^ Across nearly all states, mortality rates increased 2- to 4-fold in the post-2020 period, reflecting the ongoing escalation in drug- and medication-related deaths. The national pattern mirrors trends reported in the broader US population, with the COVID-19 pandemic amplifying preexisting substance use and overdose crises.^[21,22]^

The controversial dominance of excess mortality in rural communities followed by urban areas points to the changing drug supply dynamics and indicates the need for risk reduction interventions that are specific to community contexts. The high proportion of home deaths as opposed to hospital/medical system-based deaths similarly reflects missed chances for early intervention and further highlights the pressing need for more widespread naloxone distribution, telehealth-based addiction support, and community response. The observed state-level heterogeneity likely reflects differences in prescribing practices, drug availability (including synthetic opioids), socioeconomic vulnerability, and access to addiction treatment services. The post-2020 acceleration in nearly all jurisdictions supports evidence that pandemic-related stressors, disrupted healthcare access, and increased synthetic opioid availability significantly contributed to excess mortality.^[23]^

The results of death trends at various places are consistent with national surveillance data that demonstrate deaths from drugs and medications among older adults increasingly take place in private homes, often prior to ambulance transport. The higher rates of home death may be explained by greater access to prescription drugs, polypharmacy, and lack of supervision on the use of medications for the elderly living in the community.^[22,23]^

Collectively, these results support that drug-poisoning accidents involve a complex public health crisis with structural, social, and economic determinants. Prevention needs to be more than just pharmacologic treatment and extend into the realms of mental health service integration, poverty reduction, and stigma mitigation, addressing the causes that allow this burden of mortality to continue. Ongoing surveillance using CDC WONDER and state-based data is critical for monitoring changes in the trajectory of overdose mortality over time, as well as assessing the impact of public health policies and harm reduction initiatives.

### 4.1. Limitations

This study has several limitations. First, it has been entirely dependent on data from death certificates in the CDC WONDER database, which are subject to misclassification and underreporting, particularly regarding substances or intent (e.g., whether poisonings were accidental vs undetermined). Differences among states or with time in toxicology testing practices and encoding of cause of death may have affected estimates if they differ across strata. Second, confounding by comorbid mental disorders, socioeconomic variables, and health service access parameters is not considered, restricting causal inferences. Causality cannot be inferred from this analysis. Death certificate data identify substances involved in fatal poisoning events based on certifier judgment and coding conventions, without confirmation of dose, exposure timing, or mechanistic pathways. Consequently, findings should be interpreted as population-level associations rather than evidence of direct causal effects of specific drugs or biological substances. Finally, though JPR does a good job in detecting changes in trend, it does not adjust for unmeasured confounders or nonlinear effects. Despite these limitations, the use of national representative data over a period of more than 2 decades offers a strong depiction of the changing epidemiology of drug-related accidental poisonings in the United States. Moreover, this study intentionally avoids political attribution, as mortality surveillance data cannot support causal linkage between poisoning-related deaths and specific governmental administrations or policy decisions. Our findings are consistent with prior national surveillance reports documenting sustained increases in accidental poisoning mortality in the United States, particularly since the early 2000s. Similar upward trends and demographic disparities have been reported by CDC analyses and other population-based studies. However, our study extends existing literature by providing a longer temporal scope and detailed stratification across sex, race, geographic region, and place of death.

## 5. Conclusion

In conclusion, this analysis of CDC WONDER mortality data from 1999 through 2023 demonstrates an ongoing and pronounced increase in the number of deaths from accidental poisoning and exposure to noxious substances involving drugs, medicaments, and biological agents among all demographic groups in the United States. The report continues to reinforce that drug overdose deaths are not confined to any single population but rather reflect an increasing and pervasive public health emergency exacerbated by the COVID-19 pandemic, escalation of synthetic opioids, and longstanding socioeconomic and racial inequalities. The changing patterns we have seen, including the recent rises in NH Black and American Indian/Alaska Native populations, as well as the transition of increased mortality from rural to urban areas, highlight the need for interventions and comprehensive prevention efforts based on accurate data. Building, maintaining, and expanding more equitable addiction treatment access as well as surveillance systems, improving medication safety in older adults, and addressing the structural determinants of health will be necessary to reverse these trends and prevent similar future losses.

## Author contributions

**Conceptualization:** Sujata Lodh, Sumia Fatima, Rida Noor.

**Data curation:** Sujata Lodh.

**Project administration:** Muddassir Khalid.

**Supervision:** Muddassir Khalid.

**Validation:** Amisha Kumari.

**Visualization:** Amisha Kumari.

**Writing – original draft:** Sujata Lodh.

**Writing – review & editing:** Muddassir Khalid.

## Supplementary Material
