# Supplementary material for: Trends in accidental poisoning and exposure to noxious substances involving drugs, medicaments, and biological substances–related deaths: A nationwide US analysis, 1999 to 2023
Source: Medicine (Baltimore). 2026 May 1;105(18):e48537. doi: 10.1097/MD.0000000000048537 (PMC13138426; doi:10.1097/MD.0000000000048537)
Supplement: Supplementary file 1 [file medi-105-e48537-s001.pdf]

Graphical Abstract:

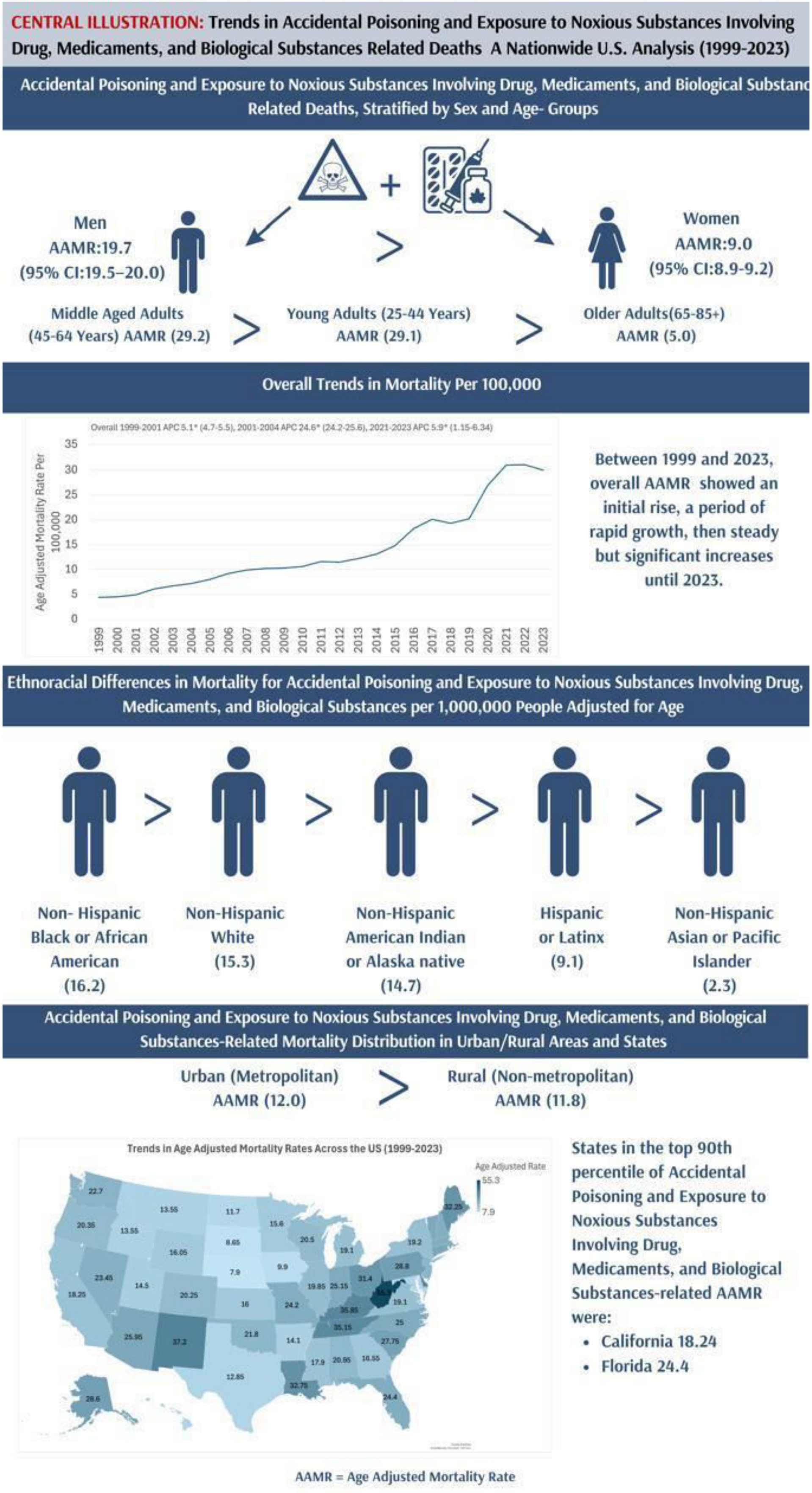

Supplementary Table 1: ***Annual Deaths from Accidental Poisoning and Exposure to Noxious Substances Involving Drugs, Medicaments, and Biological Substances, Stratified by Sex and Race in the United States, 1999–2023.***

Year	Overall	Female	Male	NH White	NH Black or African American	NH Asian or Pacific Islander	NH American Indian or Alaska Native	Hispanic or Latino
**1999**	13313	3804	9509	10612	2497	94	110	1619
**2000**	13955	4132	9823	11315	2438	85	117	1421
**2001**	15201	4598	10603	12386	2587	106	122	1410
**2002**	18948	6119	12829	15815	2829	127	177	1767
**2003**	21092	6939	14153	17821	2912	143	216	1948
**2004**	22747	7710	15037	19241	3091	157	258	1837
**2005**	25496	8404	17092	21506	3513	192	285	2195
**2006**	29528	9721	19807	24885	4124	220	299	2455
**2007**	30336	10558	19778	26165	3637	225	309	2328
**2008**	30788	10872	19916	27015	3190	216	367	2370
**2009**	31595	11393	20202	27791	3139	246	419	2472
**2010**	32950	12185	20765	29143	3175	244	388	2453
**2011**	36129	13280	22849	31847	3537	307	438	2819
**2012**	36325	13365	22960	31902	3599	332	492	2933
**2013**	38872	14083	24789	34020	3996	383	473	3277
**2014**	42013	15139	26874	36614	4399	429	571	3447

**2015**	47644	16323	31321	41419	5097	525	603	4042
**2016**	58736	18932	39804	50136	7287	627	686	5164
**2017**	65570	20700	44870	55190	8889	732	759	6019
**2018**	63371	19749	43622	52586	9219	769	797	6420
**2019**	66986	20135	46851	54544	10672	858	912	7558
**2020**	88944	25971	62973	70677	15755	1229	1283	10746
**2021**	104476	30520	73956	80714	19575	943	1572	13211
**2022**	105497	29988	75509	79509	21323	1098	1749	14295
**2023**	103479	28897	74582	76123	22471	1115	1816	14762
**Total**	1118830	363517	780474	938976	172951	11402	15218	118968

NH= non-Hispanic; N/A=not available (unreliable or suppressed)

Supplementary Table 2: ***Annual Deaths from Accidental Poisoning and Exposure to Noxious Substances Involving Drugs, Medicaments, and Biological Substances, Stratified by Place of Death Among Older Adults in the United States, 1999–2023.***

Place of Death	Deaths (1999-2023)	Percentage
Medical Facility - Inpatient	113546	9.95%
Medical Facility - Outpatient or ER	153082	12.95%
Medical Facility - Dead on Arrival	22329	1.60%
Medical Facility - Status unknown	222	0.00%
Total (Medical Facility)	289179	27.40%
Decedent's home	565384	50.15%
Hospice facility	2726	0.25%
Nursing home/long term care	2982	0.25%
Other	255978	24.65%
Place of death unknown	2581	0.15%
	1118830	100.00%

N/A= not available (unreliable or suppressed)

Supplementary Table 3: ***Annual Percent Change (APC) in Age-Adjusted Mortality Rates per 100,000 Due to Accidental Poisoning and Exposure to Noxious Substances Involving Drugs, Medicaments, and Biological Substances in the United States, 1999–2023.***

Year Interval	APC (95% CI)	P value
**Overall**		
1999-2001	5.11* (4.74 – 5.50)	< 0.000001
2001-2004	24.85* (24.15 – 25.62)	< 0.000001
2004-2023	5.87* (1.15 – 6.34)	0.026795
**Male**		
1999-2013	5.41 (–1.14 – 7.90)	0.067986
2013-2023	11.47* (9.43 – 17.10)	0.0008
**Female**		
1999-2023	11.05* (9.71 – 12.30)	< 0.000001
**NH White**		
1999-2001	7.38* (7.02–7.74)	< 0.000001
2001-2023	8.95 (–15.56–33.18)	0.116377
**NH Black or African American**		
1999-2013	0.97 (–1.81 – 3.45)	0.34753
2013-2023	19.52* (17.03 – 23.18)	< 0.000001
**NH American Indian or Alaska Native**		
1999-2004	14.96* (8.24 – 41.23)	0.0012
2004-2018	5.66* (2.53 – 6.63)	0.023595
2018-2021	30.59* (24.38 – 35.07)	0.002

2021-2023	8.15* (2.33 – 14.81)	0.009598
**NH Asian or Pacific Islander**		
1999-2013	5.54* (0.92–7.98)	0.038392
2013-2020	12.16* (10.01–19.55)	0.015597
2020-2023	3.78 (–1.78–8.53)	0.131974
**Hispanic or Latino**		
1999-2013	1.68 (–2.17 – 5.18)	0.14917
2013-2018	13.11 (–2.10 – 16.30)	0.065987
2018-2021	23.54* (16.53 – 27.79)	0.014397
2021-2023	3.93 (–2.35 – 14.00)	0.118376
**Rural areas**		
1999-2006	17.77* (13.87 – 23.29)	0.004399
2006-2013	4.99* (1.20 – 6.34)	0.031994
2013-2020	16.00* (6.42 – 21.48)	< 0.000001
**Urban areas**		
1999-2006	12.23* (9.24 – 24.89)	0.0008
2006-2013	4.43 (–2.28 – 6.71)	0.191962
2013-2020	11.01* (9.23 – 16.38)	0.0008
**Northeast**		
1999-2013	7.4(0.4-10.1)	0.045191
2013-2016	25.6*(12.4-32.2)	0.007199
2016-2023	3.9*(-0.2-6.1)	0.057588

**Midwest**		
1999-2021	9.99*(9.45-0.0)	0.0012
2021-2023	-3.4(-11.4-0.0)	0.588682
**South**		
1999-2015	5.56 (–2.04 – 8.43)	0.081184
2015-2023	11.78* (8.41 – 20.22)	0.007199
**West**		
1999-2008	7.17* (5.35 – 11.73)	0.004799
2008-2018	2.63* (0.05 – 3.72)	0.04799
2018-2021	26.03* (20.16 – 29.87)	0.003999
2021-2023	4.98 (–1.25 – 12.18)	0.104379

Supplementary Table 4: ***Overall and Sex-Stratified Age-Adjusted Mortality Rates per 100,000 Due to Accidental Poisoning and Exposure to Noxious Substances Involving Drugs, Medicaments, and Biological Substances in the United States, 1999–2023.***

	Age-Adjusted Rate (95% CI)		
**Year**	**Male**	**Female**	**Overall**
**1999**	6.9 (6.8-7.1)	2.6 (2.6-2.7)	4.8 (4.7-4.9)
**2000**	7.1 (6.9-7.2)	2.8 (2.7-2.9)	5 (4.9-5)
**2001**	7.5 (7.4-7.7)	3.2 (3.1-3.3)	5.3 (5.2-5.4)
**2002**	9.1 (8.9-9.2)	4.2 (4.1-4.3)	6.6 (6.5-6.7)
**2003**	9.9 (9.7-10.1)	4.7 (4.6-4.8)	7.3 (7.2-7.4)
**2004**	10.4 (10.2-10.6)	5.2 (5.1-5.3)	7.8 (7.7-7.9)
**2005**	11.7 (11.5-11.9)	5.6 (5.5-5.7)	8.6 (8.5-8.7)
**2006**	13.4 (13.2-13.6)	6.4 (6.3-6.5)	9.8 (9.7-10)
**2007**	13.2 (13.1-13.4)	6.8 (6.7-6.9)	10 (9.9-10.1)

**2008**	13.2 (13-13.4)	6.9 (6.8-7.1)	10.1 (9.9-10.2)
**2009**	13.3 (13.1-13.4)	7.2 (7.1-7.3)	10.2 (10.1-10.3)
**2010**	13.5 (13.3-13.7)	7.7 (7.5-7.8)	10.6 (10.5-10.7)
**2011**	14.8 (14.6-15)	8.3 (8.2-8.5)	11.5 (11.4-11.7)
**2012**	14.8 (14.6-14.9)	8.3 (8.2-8.4)	11.5 (11.4-11.6)
**2013**	15.8 (15.6-16)	8.7 (8.5-8.8)	12.2 (12.1-12.4)
**2014**	17 (16.8-17.2)	9.3 (9.1-9.4)	13.1 (13-13.2)
**2015**	19.7 (19.5-20)	10 (9.8	14.8 (14.7-15)
**2016**	25 (24.8-25.3)	11.6 (11.4-11.8)	18.3 (18.2-18.5)
**2017**	28 (27.7-28.3)	12.7 (12.5-12.8)	20.3 (20.1-20.5)
**2018**	27.1 (26.8-27.4)	12 (11.8-12.2)	19.5 (19.4-19.7)

**2019**	29 (28.7-29.2)	12.2 (12-12.4)	20.6 (20.4-20.7)
**2020**	39 (38.7-39.3)	15.8 (15.6-16)	27.4 (27.2-27.5)
**2021**	44.8 (44.5-45.1)	18.5 (18.3-18.7)	31.6 (31.4-31.8)
**2022**	45.3 (45-45.7)	18.2 (18-18.4)	31.8 (31.6-32)
**2023**	44.4 (44.1-44.7)	17.3 (17-17.5)	30.8 (30.6-31)
**Overall**	19.7 (19.5–20.0)	9.0 (8.9-9.2)	14.4 (14.3-14.5)

Supplementary Table 5: Accidental Poisoning and Exposure to Noxious Substances Involving Drug, Medicaments, and Biological Substances Related Age-Adjusted Mortality Rates per 100,000, Stratified by Race in the United States, 1999 to 2023

	Age-Adjusted Rate (95% CI)				
**Year**	**NH White**	**NH Black or African American**	**NH American Indian or Alaska Native**	**NH Asian or Pacific Islander**	**Hispanic or Latino**
**1999**	4.6 (4.5–4.7)	7.6 (7.3–7.9)	4.2 (3.4–5.1)	0.9 (0.7–1.1)	5.5 (5.3–5.8)
**2000**	4.9 (4.8–5.0)	7.3 (7.0–7.6)	4.3 (3.5–5.2)	0.7 (0.6–0.9)	5.6 (5.4–5.8)
**2001**	5.3 (5.2–5.4)	7.6 (7.3–7.9)	4.2 (3.5–5.0)	0.9 (0.7–1.0)	5.4 (5.1–5.6)
**2002**	6.8 (6.7–6.9)	8.1 (7.8–8.5)	5.7 (4.9–6.6)	1.0 (0.8–1.2)	5.9 (5.7–6.1)
**2003**	7.6 (7.5–7.7)	8.2 (7.9–8.5)	7.1 (6.1–8.0)	1.0 (0.9–1.2)	6.1 (5.9–6.3)
**2004**	8.1 (8.0–8.3)	8.6 (8.2–8.9)	7.9 (6.9–8.9)	1.1 (1.0–1.3)	6.6 (6.4–6.8)
**2005**	9.0 (8.9–9.2)	9.5 (9.2–9.8)	8.6 (7.6–9.6)	1.4 (1.2–1.6)	6.7 (6.4–6.9)

**2006**	10.4 (10.2–10.5)	10.9 (10.6–11.2)	8.7 (7.7–9.8)	1.5 (1.3–1.7)	7.6 (7.3–7.8)
**2007**	10.8 (10.7–10.9)	9.4 (9.1–9.7)	8.6 (7.6–9.6)	1.4 (1.2–1.6)	9.4 (9.1–9.6)
**2008**	11.1 (10.9–11.2)	8.1 (7.9–8.4)	9.8 (8.7–10.8)	1.3 (1.1–1.5)	10.7 (10.4–11.0)
**2009**	11.3 (11.2–11.5)	7.8 (7.5–8.1)	10.7 (9.7-11.8)	1.5 (1.3–1.7)	11.2 (10.9–11.5)
**2010**	11.9 (11.7–12.0)	7.8 (7.5–8.0)	9.6 (8.6–10.5)	1.4 (1.2–1.6)	12.9 (12.6–13.2)
**2011**	12.9 (12.8–13.1)	8.5 (8.2–8.8)	10.6 (9.6–11.6)	1.7 (1.5–1.9)	17.9 (17.6–18.3)
**2012**	12.9 (12.7–13.0)	8.5 (8.2–8.8)	11.6 (10.6–12.7)	1.8 (1.6–2.0)	21.5 (21.2–21.9)
**2013**	13.7 (13.5–13.8)	9.3 (9.0–9.6)	11.1 (10.1–12.2)	2.0 (1.8–2.2)	23.0 (22.6–23.4)
**2014**	14.7 (14.6–14.9)	10.1 (9.8–10.4)	13.3 (12.2–14.4)	2.1 (1.9–2.3)	23.2 (22.8–23.6)
**2015**	16.7 (16.5–16.8)	11.5 (11.2–11.8)	13.7 (12.6–14.8)	2.5 (2.3–2.7)	18.5 (18.1–18.8)
**2016**	20.3 (20.1–20.5)	16.2 (15.8–16.5)	15.3 (14.1–16.5)	2.9 (2.6–3.1)	3.5 (3.4–3.7)

**2017**	22.3 (22.1–22.5)	19.4 (19.0–19.9)	16.8 (15.6–18.0)	3.2 (3.0–3.5)	5.5 (5.3–5.8)
**2018**	21.2 (21.0–21.4)	19.9 (19.5–20.3)	17.5 (16.2–18.7)	3.4 (3.1–3.6)	5.6 (5.4–5.8)
**2019**	22.0 (21.8–22.2)	22.8 (22.4–23.3)	19.5 (18.2–20.8)	3.7 (3.4–3.9)	5.4 (5.1–5.6)
**2020**	28.6 (28.4–28.9)	33.4 (32.9-34.0)	27.1 (25.6–28.6)	5.2 (4.9–5.5)	5.9 (5.7–6.1)
**2021**	32.7 (32.5–33.0)	42.2 (41.6–42.8)	37.0 (35.2–38.9)	4.4 (4.1–4.6)	6.1 (5.9–6.3)
**2022**	32.2 (32.0–32.5)	45.8 (45.1–46.4)	41.4 (39.4-43.3)	4.9 (4.6–5.2)	6.6 (6.4–6.8)
**2023**	30.5 (30.3–30.7)	47.5 (46.9–48.2)	42.0 (40.1–44.0)	4.9 (4.6–5.2)	6.7 (6.4–6.9)
**Overall**	15.3 (15.1-15.5)	15.8 (15.5-16.2)	14.7 (13.5-15.8)	2.3 (2.1-2.5)	9.1 (8.8-9.4)

Supplementary Table 6: ***Accidental Poisoning and Exposure to Noxious Substances Involving Drugs, Medicaments, and Biological Substances: Age-Adjusted Mortality Rates per 100,000, Stratified by State in the United States, 1999–2023.***

State	Age-Adjusted Rate (95%CI)	
	**1999-2020**	**2021-2023**
Alabama	10.6 (10.4–10.9)	31.3 (30.3–32.2)
Alaska	15.7 (15–16.3)	41.5 (38.7–44.2)
Arizona	15.7 (15.5–16)	36.2 (35.3–37.0)
Arkansas	8.2 (7.9–8.4)	20.0 (19.0–20.9)
California	9.7 (9.7–9.8)	26.8 (26.5–27.1)
Colorado	12.1 (11.9–12.3)	28.4 (27.6–29.2)
Connecticut	15.4 (15.1–15.7)	38.4 (37.2–39.6)
Delaware	17.9 (17.3–18.5)	52.5 (49.7–55.2)
District of Columbia	19.3 (18.6–20)	61.6 (58.2–65.0)
Florida	15 (14.9–15.2)	33.8 (33.4–34.3)
Georgia	9.6 (9.5–9.7)	23.5 (22.9–24.0)
Hawaii	8.1 (7.7–8.4)	17.5 (16.2–18.7)
Idaho	8.7 (8.4–9)	18.4 (17.2–19.5)
Illinois	11.8 (11.6–11.9)	27.9 (27.4–28.4)
Indiana	13 (12.9–13.2)	37.3 (36.4–38.1)
Iowa	6.3 (6.1–6.5)	13.5 (12.8–14.3)
Kansas	8.4 (8.2–8.6)	23.6 (22.6–24.7)
Kentucky	20.5 (20.2–20.8)	51.2 (49.9–52.4)
Louisiana	14.6 (14.4–14.8)	50.9 (49.7–52.1)

Maine	14.7 (14.3–15.2)	49.8 (47.5–52.0)
Maryland	4.4 (4.3–4.5)	20.9 (20.3–21.6)
Massachusetts	14.5 (14.3–14.6)	34.7 (33.9–35.5)
Michigan	11.8 (11.7–12)	26.4 (25.8–27.0)
Minnesota	7.6 (7.4–7.7)	23.6 (22.8–24.4)
Mississippi	9.3 (9.1–9.5)	26.5 (25.4–27.7)
Missouri	14 (13.8–14.2)	34.4 (33.5–35.3)
Montana	8.6 (8.2–9)	18.5 (17.0–20.0)
Nebraska	5.3 (5.1–5.5)	10.5 (9.6–11.4)
Nevada	16.2 (15.8–16.5)	30.7 (29.6–31.8)
New Hampshire	15.5 (15.1–16)	33.0 (31.2–34.9)
New Jersey	13.6 (13.5–13.8)	30.4 (29.8–31.1)
New Mexico	22.3 (21.8–22.7)	52.1 (50.2–53.9)
New York	9.6 (9.5–9.7)	28.8 (28.4–29.3)
North Carolina	12.9 (12.8–13.1)	37.1 (36.4–37.8)
North Dakota	5.5 (5.1–5.8)	17.9 (16.1–19.7)
Ohio	18.6 (18.5–18.8)	44.2 (43.4–44.9)
Oklahoma	14.9 (14.6–15.2)	28.7 (27.7–29.7)
Oregon	9.3 (9.1–9.5)	31.4 (30.4–32.4)
Pennsylvania	18.8 (18.6–19)	38.8 (38.2–39.5)
Rhode Island	15.5 (15–16.1)	38.4 (36.2–40.6)
South Carolina	13.2 (13–13.4)	42.3 (41.2–43.3)
South Dakota	5.4 (5.1–5.8)	11.9 (10.6–13.3)

Tennessee	16.5 (16.2–16.7)	53.8 (52.8–54.8)
Texas	8.4 (8.3–8.4)	17.3 (17.0–17.6)
Utah	10.9 (10.6–11.2)	18.1 (17.2–18.9)
Vermont	11.1 (10.5–11.7)	40.4 (37.4–43.4)
Virginia	9.7 (9.6–9.9)	28.5 (27.8–29.2)
Washington	11.8 (11.7–12)	33.6 (32.9–34.4)
West Virginia	27.3 (26.8–27.8)	83.3 (80.7–85.9)
Wisconsin	11 (10.9–11.2)	30.0 (29.2–30.9)
Wyoming	11 (10.4–11.6)	21.1 (18.8–23.3)

N/A = not available (unreliable or suppressed)

Supplementary Table 7: Accidental Poisoning and Exposure to Noxious Substances Involving Drug, Medicaments, and Biological Substances Related Age-Adjusted Mortality Rates per 100,000, Stratified by Census Region in the United States, 1999 to 2023

	Age-Adjusted Rate (95% CI)			
**Year**	**Northeast**	**Midwest**	**South**	**West**
**1999**	4.5 (4.3–4.7)	3.3 (3.2–3.5)	3.7 (3.5–3.8)	3.7 (3.5–3.8)
**2000**	4.8 (4.6–5.0)	3.8 (3.6–3.9)	4.3 (4.2–4.4)	4.3 (4.2–4.4)
**2001**	5.1 (4.9–5.3)	4.2 (4.0–4.3)	5.5 (5.4–5.7)	5.5 (5.4–5.7)
**2002**	5.6 (5.4–5.8)	5.0 (4.8–5.2)	6.3 (6.2–6.5)	6.3 (6.2–6.5)
**2003**	6.1 (5.9–6.3)	5.0 (4.8–5.2)	7.5 (7.3–7.6)	7.5 (7.3–7.6)
**2004**	6.0 (5.8–6.2)	6.2 (6.0–6.4)	7.9 (7.7–8.1)	7.9 (7.7–8.1)
**2005**	7.7 (7.5–7.9)	7.0 (6.8–7.2)	8.5 (8.3–8.7)	8.5 (8.3–8.7)
**2006**	9.5 (9.3–9.8)	8.6 (8.4–8.8)	9.6 (9.4–9.8)	9.6 (9.4–9.8)
**2007**	9.5 (9.3–9.8)	9.0 (8.8–9.2)	10.3 (10.1–10.5)	10.3 (10.1–10.5)
**2008**	9.7 (9.5–10.0)	9.7 (9.4–9.9)	10.5 (10.3–10.6)	10.5 (10.3–10.6)
**2009**	8.8 (8.6–9.0)	9.8 (9.5–10.0)	10.9 (10.7–11)	10.9 (10.7–11.0)
**2010**	9.6 (9.3–9.8)	10.4 (10.2–10.7)	11.2 (11–11.4)	11.2 (11.0–11.4)
**2011**	11.5 (11.3–11.8)	11.5 (11.3–11.8)	11.8 (11.6–12)	11.8 (11.6–12.0)
**2012**	12.4 (12.1–12.7)	12.1 (11.8–12.3)	11.1 (10.9–11.3)	11.1 (10.9–11.3)

**2013**	13.7 (13.4–14.0)	13.2 (12.9–13.5)	11.4 (11.2–11.6)	11.4 (11.2–11.6)
**2014**	15.2 (14.9–15.6)	14.6 (14.3–14.9)	12.2 (12–12.4)	12.2 (12.0–12.4)
**2015**	18.8 (18.4–19.2)	16.4 (16.0–16.7)	13.7 (13.5–13.9)	13.7 (13.5–13.9)
**2016**	25.5 (25.1–26.0)	20.8 (20.5–21.2)	16.9 (16.7–17.2)	16.9 (16.7–17.2)
**2017**	28.7 (28.2–29.1)	23.9 (23.5–24.3)	18.4 (18.2–18.7)	18.4 (18.2–18.7)
**2018**	27.3 (26.8–27.7)	21.7 (21.4–22.1)	17.5 (17.2–17.7)	17.5 (17.2–17.7)
**2019**	27.0 (26.5–27.4)	22.5 (22.2–22.9)	18.6 (18.3–18.8)	18.6 (18.3–18.8)
**2020**	31.8 (31.3–32.3)	28.7 (28.3–29.1)	26.6 (26.3–26.9)	26.6 (26.3–26.9)
**2021**	34.2 (33.7–34.7)	31.4 (31.0–31.8)	31.4 (31.1–31.7)	31.4 (31.1–31.7)
**2022**	34.4 (33.9–34.9)	31.0 (30.5–31.4)	31.7 (31.4–32)	31.7 (31.4–32.0)
**2023**	31.9 (31.4–32.4)	28.0 (27.6–28.4)	29.9 (29.6–30.2)	29.9 (29.6–30.2)

**Overall**	23.55 (23.25-23.85)	21.17 (20.90-21.37)	21.45 (21.25-21.60)	19.95 (19.72 – 20.13)

Supplementary Table 8: Accidental Poisoning and Exposure to Noxious Substances Involving Drugs, Medicaments, and Biological Substances: Age-Adjusted Mortality Rates per 100,000 in the United States Stratified by Urban–Rural Classification, 1999–2020

	Age-Adjusted Rate (95% CI)	
**Year**	**Urban**	**Rural**
1999	4.2(4.1–4.4)	3.1(2.8–3.3)
2000	4.4(4.3–4.6)	3.5(3.3–3.8)
2001	4.9(4.7–5.1)	4.4(4.1–4.7)
2002	6.0(5.9–6.2)	5.5(5.1–5.8)
2003	6.7(6.4–6.9)	6.7(6.4–7.1)
2004	7.1(6.9–7.4)	7.6(7.2–7.9)
2005	8.0(7.7–8.2)	8.1(7.7–8.4)
2006	9.1(8.9–9.4)	9.5(9.0–9.9)
2007	9.8(9.6–10.0)	10.5(10.1–11.0)
2008	10.1(9.9–10.4)	11.2(10.7–11.6)
2009	10.3(10.0–10.6)	11.6(11.2–12.1)
2010	10.7(10.4–10.9)	12.2(11.7–12.7)
2011	11.6(11.3–11.9)	13.2(12.8–13.8)
2012	11.6(11.3–11.9)	13.0(12.5–13.5)
2013	12.4(12.1–12.7)	13.0(12.5–13.5)
2014	13.3(13.1–13.7)	14.2(13.7–14.8)
2015	15.1(14.7–15.4)	15.4(14.9–16.0)
2016	18.5(18.2–18.9)	17.1(16.6–17.7)
2017	20.4(20.1–20.8)	18.4(17.8–19.0)

2018	19.6(19.2–19.9)	17.7(17.1–18.2)
2019	20.4(20.1–20.8)	18.4(17.8–19.0)
2020	27.3(26.8–27.7)	25.1(24.4–25.8)
Overall	11.9(11.6-12.2)	11.8(11.3–12.2)
